# Adolescent Vulnerability to Heightened Emotional Reactivity and Anxiety After Brief Exposure to an Obesogenic Diet

**DOI:** 10.3389/fnins.2020.00562

**Published:** 2020-06-30

**Authors:** Julio D. Vega-Torres, Matine Azadian, Raul A. Rios-Orsini, Arsenio L. Reyes-Rivera, Perla Ontiveros-Angel, Johnny D. Figueroa

**Affiliations:** ^1^Physiology Division, Department of Basic Sciences, Center for Health Disparities and Molecular Medicine, Loma Linda University School of Medicine, Loma Linda, CA, United States; ^2^Stanford University School of Medicine, Stanford, CA, United States; ^3^San Juan Bautista School of Medicine, Caguas, Puerto Rico

**Keywords:** PTSD, diet-induced obesity, adolescence, anxiety, fear-potentiated startle, CRHR1

## Abstract

**Background:**

Emerging evidence demonstrates that diet-induced obesity disrupts corticolimbic circuits underlying emotional regulation. Studies directed at understanding how obesity alters brain and behavior are easily confounded by a myriad of complications related to obesity. This study investigated the early neurobiological stress response triggered by an obesogenic diet. Furthermore, this study directly determined the combined impact of a short-term obesogenic diet and adolescence on critical behavioral and molecular substrates implicated in emotion regulation and stress.

**Methods:**

Adolescent (postnatal day 31) or adult (postnatal day 81) Lewis rats were fed for 1 week with an experimental Western-like high-saturated fat diet (*WD*, 41% kcal from fat) or a matched control diet (*CD*, 13% kcal from fat). We used the acoustic fear-potentiated startle (FPS) paradigm to determine the effects of the WD on cued fear conditioning and fear extinction. We used c-Fos mapping to determine the functional influence of the diet and stress on corticolimbic circuits.

**Results:**

We report that 1-week WD consumption was sufficient to induce fear extinction deficits in adolescent rats, but not in adult rats. We identify fear-induced alterations in corticolimbic neuronal activation and demonstrate increased prefrontal cortex CRHR1 messenger RNA (mRNA) levels in the rats that consumed the WD.

**Conclusion:**

Our findings demonstrate that short-term consumption of an obesogenic diet during adolescence heightens behavioral and molecular vulnerabilities associated with risk for anxiety and stress-related disorders. Given that fear extinction promotes resilience and that fear extinction principles are the foundation of psychological treatments for posttraumatic stress disorder (PTSD), understanding how obesogenic environments interact with the adolescent period to affect the acquisition and expression of fear extinction memories is of tremendous clinical relevance.

## Highlights

– Short-term WD consumption during adolescence impairs cued fear extinction memory retention in a fear-potentiated startle paradigm.
– Short-term WD consumption during adolescence attenuates neuronal activation to electric footshock stress in the basomedial nuclei of the amygdala.
– Short-term WD consumption increases CRHR1 mRNA levels in the medial prefrontal cortex.
– Adult LEW rats exhibit increased basal HPA axis tone and heightened emotional reactivity to footshock stress relative to adolescent rats.

## Introduction

Early-life trauma is linked to obesity and the consumption of obesogenic diets (Perkonigg et al., 2009; Pagoto et al., 2012; Roenholt et al., 2012; Ehlert, 2013; Duncan et al., 2015; Farr et al., 2015; Masodkar et al., 2016; Mason et al., 2017; Wolf et al., 2017). The high comorbidity between obesity and posttraumatic stress disorders (PTSDs) suggest that adaptations to trauma may increase the risk for the consumption of obesogenic diets as a result of the traumatic experience (Kalyan-Masih et al., 2016; Michopoulos et al., 2016; Godfrey et al., 2018). There is also mounting evidence that exposure to obesogenic diets rich in saturated fat foods and sugars have a direct adverse effect on emotional regulation, anxiety-like behaviors, and neural substrates implicated with stress (Ortolani et al., 2011; Boitard et al., 2015; Reichelt et al., 2015; Sivanathan et al., 2015; Baker and Reichelt, 2016; Kalyan-Masih et al., 2016; Vega-Torres et al., 2018). Therefore, early-life exposure to obesogenic diets may predispose individuals to maladaptive stress responses, resulting in increased PTSD risk.

Several lines of evidence suggest that alterations in attention, memory, and learning contribute to the etiology and maintenance of PTSD symptoms (Lissek and van Meurs, 2015; Liberzon and Abelson, 2016). Interestingly, while fear learning emerges early in life, fear memories undergo dynamic changes during adolescence (King et al., 2014; Baker and Richardson, 2015; Ganella et al., 2017). Studies indicate that extinction learning is blunted during adolescence (Pattwell et al., 2012), which has important implications for PTSD treatment. The fear-potentiated startle (FPS) represents a proven and reliable method for examining conditioned fear responses (Davis et al., 1993). This method shows notable face validity, construct validity, and predictive validity in the assessment of behaviors and circuits implicated in PTSD. The highly conserved corticolimbic circuit is critical for cue-elicited fear responses and safety learning (Likhtik et al., 2014; Likhtik and Paz, 2015). In particular, the corticolimbic pathway connecting the medial prefrontal cortex (mPFC) and the amygdala undergoes dramatic structural reorganization during adolescence (Casey, 2015; Arruda-Carvalho et al., 2017; Silvers et al., 2017) and remains the focus of our recent investigations. Our studies indicate that this corticolimbic pathway is highly vulnerable to the disruptive effects of an obesogenic diet when exposure to the diet starts during adolescence (Vega-Torres et al., 2018). In that study, we demonstrated that Western-like high-saturated fat diet (WD) consumption during the critical period of adolescence leads to increased anxiety-like behaviors. Using the fear-potentiated startle paradigm, we found significant deficits in fear learning and fear extinction learning in the rats that consumed the WD during adolescence. Furthermore, we identified new structural impairments induced by the WD, particularly in brain regions related to fear and anxiety processing. Notably, we found that WD consumption for 4 weeks during adolescence was sufficient to disrupt brain structure and behavior. However, without a cohort of animals exposed to the obesogenic diet only during adulthood in our previous studies, it is difficult to determine if adolescence represents a distinct period of vulnerability to the detrimental effects of an obesogenic diet on learned fear and emotional reactivity. Furthermore, few studies have tested the hypothesis that short-term consumption of an obesogenic diet is sufficient to alter fear responses.

This study tested several hypotheses. First, we predicted impaired fear extinction in adolescent rats that consumed an obesogenic diet for 1 week. Not only would this represent a replication of our previous report in adult rats (Vega-Torres et al., 2018), but it would also extend that finding to the effects of diet that are independent of obesity-related processes. Second, we reasoned that short-term exposure to an obesogenic diet would reduce the neuronal activation in corticolimbic regions implicated in fear extinction and anxiolytic effects. Finally, given the robust effects of obesogenic diets on the hypothalamic–pituitary–adrenal (HPA) axis and dopamine systems (Reyes, 2012; Sharma et al., 2013; Boitard et al., 2015; Khazen et al., 2019) and the modulatory actions of these factors on mPFC–amygdala circuit function (Fadok et al., 2009b; Jovanovic et al., 2010, 2020; Veer et al., 2012; Whittle et al., 2016), we hypothesized that the obesogenic diet would increase the HPA tone while reducing dopamine receptor expression in the mPFC.

This study demonstrates that short-term exposure to an obesogenic diet is sufficient to impair retention of fear extinction training while altering neurobiological substrates implicated with emotional reactivity. These findings reveal a unique interplay between high-saturated fat/high-sugar foods and fear extinction during adolescence, which may prove informative for understanding risk factors implicated in stress-related disorders. More importantly, this study suggests that obesity and the consumption of obesogenic diets may represent a mediator of differential anxiety and stress-related disorders psychotherapy treatment outcomes.

## Materials and Methods

### Animals

All the experiments were performed following protocols approved by the Institutional Animal Care and Use Committee (IACUC) at the Loma Linda University School of Medicine. Adolescent [ADOL: postnatal day (PND), 24] and adult (ADUL: PND, 74) male Lewis rats were purchased from Charles River Laboratories (Portage, MI, United States). Although the precise correlation between age of rats and humans is still controversial, it has been proposed that adolescence occurs between PND 28 and 42 in rats (Spear, 2000; Sengupta, 2013). Thus, a human year corresponds to approximately 3 days in the life of a rat (Sengupta, 2013). The rationale for the use of Lewis rats is based on the relevant vulnerabilities of this strain to posttraumatic stress (Kalyan-Masih et al., 2016; Vega-Torres et al., 2018). Immediately upon arrival, the rats were housed in groups (two per cage) with free access to food and water. Dietary manipulations commenced at PND 31 for adolescent rats and at PND 81 for adult rats. Animals were kept in customary housing conditions (21 ± 2°C, relative humidity of 45%, and 12-h light/dark cycle with lights on at 7:00 AM). The body weights were recorded once a week or daily during the week of behavioral testing. Food consumption was quantified at least twice per week. The rats were never food or water restricted.

### Diets

The standard chow diet (CHOW, 4 g% fat, product no. 7001) was obtained from Teklad Diets (Madison, WI, United States), while the matched low-fat purified control diet (CD, 5 g% fat, product no. F7463) and Western-like high-saturated fat diet (WD, 20 g% fat, product no. F7462) were obtained from Bio-Serv (Frenchtown, NJ, United States). There is an increasing awareness of the impact of diet changes on stress reactivity (Sharma et al., 2013; Vega-Torres et al., 2018) and a need for using adequate matched diets in diet-induced obesity research (Pellizzon and Ricci, 2018). Thus, we decided to incorporate a matched low-fat control diet group along with the standard chow diet group. Research WD used to generate obesogenic phenotypes are generally high in fat and refined carbohydrates. More importantly, these diets are typically manufactured with purified ingredients. Unfortunately, several diet-induced obesity studies still report the use of grain-based chow diets as the control diet. Grain-based chow diets contain unrefined ingredients and have marked differences in the content and composition of various nutrients (e.g., fiber). Control diet choice can thus conceivably confound data interpretation and affect reproducibility. Given that initially both the CHOW and CD groups had identical biometric and behavioral outcomes, we opted to use the more appropriate CD group as control for the WD group (Vega-Torres et al., 2019). The macronutrient composition and fatty acid profiles are detailed in Table 1.

**TABLE 1:** Detailed composition of the purified diets.

	Control diet (CD) #F7463	Western diet (WD) #F7462
**Macronutrient (main source)**	**% kcal**	**% kcal**

Carbohydrates (corn starch)	68	43
Proteins (casein)	19	16
Fats (milk fat)	13	41
*Total kcal*	3.7	4.6

**Sugars**	**g/kg**	**g/kg**

Sucrose	341	340

**Fatty acid class**	**g/kg**	**g/kg**

Saturated	20.8	121
Monosaturated	12.7	492
Polyunsaturated	11.3	11.5
Ratio omega-3 to omega-6	0.02	0.01

*Dietary fat percentage was ∼5% in the CD and ∼20% in the WD. The WD contained higher levels of total saturated fatty acids (ninefold difference) and total monounsaturated fatty acids (threefold difference) when compared to the CD. The levels of total polyunsaturated fatty acids were similar between CD and WD.*

### Study Design

**FIGURE 1:**
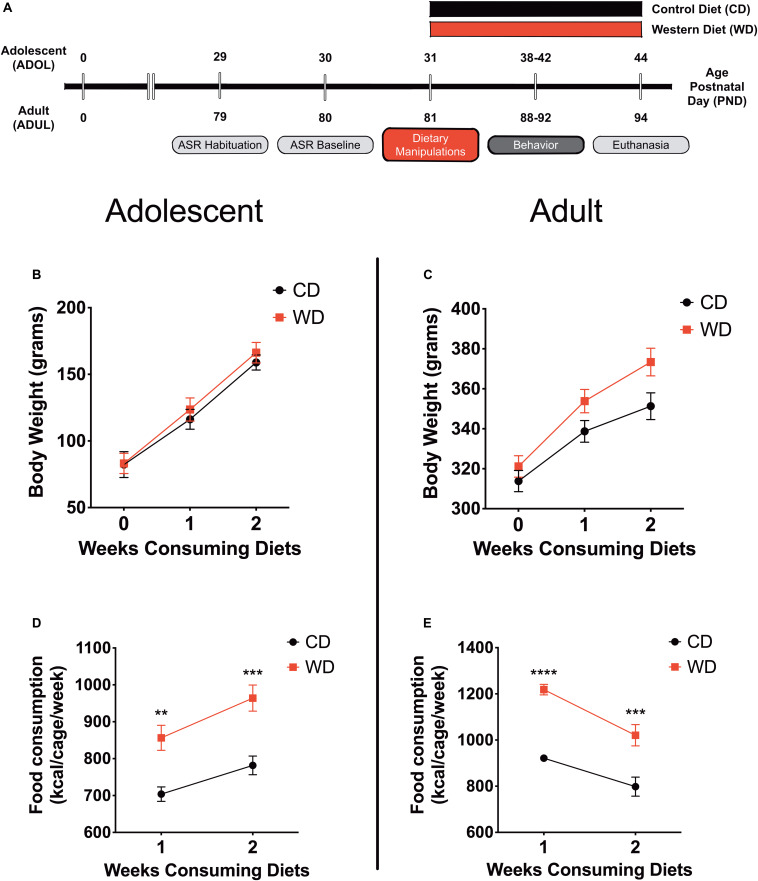
**(A)** Timeline of experimental procedures. Adolescent (*ADOL*) and adult (*ADUL*) rats were matched based on their acoustic startle reflex (ASR) responses and allocated to one of two diets: control diet (CD) or Western high-saturated fat diet (WD). The rats were exposed to the diets for 1 week before behavioral testing. A 4-day fear-potentiated startle (FPS) paradigm was used to determine the effects of the WD on cued fear conditioning and fear extinction learning. Additional anxiety-like behaviors were investigated in the elevated plus maze (EPM). All the rats were euthanized 1 day after the completion of the EPM and brains dissected for RNA extraction. Please refer to the study design in *Methods* for more specific technical details on procedures and behavioral tests performed in this study. Average weekly body weight in grams for **(B)** adolescent and **(C)** adult groups (diet effect *p* > 0.05 for both age groups; *n* = 11–12 rats/group). Average caloric intake in kilocalories per cage per week for **(D)** adolescent and **(E)** adult groups. WD groups consumed more calories than CD rats, regardless of age (ADOL: diet effect *p* = 0.002; ADUL: diet effect *p* < 0.0001; for both groups: *n* = 6 cages/group). Error bars are SEM. *p ≤ 0.05, **p ≤ 0.01, ***p ≤ 0.001, and ****p ≤ 0.0001, respectively.

Behavior testing sessions involved a 20- to 30-min acclimation period to the testing facility. Following room acclimation, the rats were placed for 5 min inside the acoustic startle reflex (ASR) enclosure and testing chamber and then returned to their cages. The next day (ADOL: PND, 30; ADUL: PND, 80), we measured baseline ASR responses, which were used to generate balanced experimental groups. The ASR-based group matching resulted in an even distribution of rats with similar startle responses in all groups. The rats were divided in the following groups: ADOL + CD (*n* = 12), ADOL + WD (*n* = 12), ADUL + CD (*n* = 12), ADUL + WD (*n* = 12). We conducted a sensitivity power analysis (Cohen, 1992) (two-way ANOVA: diet type and age as factors) using *G^∗^Power* (Faul et al., 2007). Analyses revealed that 12 rats per group are sufficient to detect medium effect sizes (*d* = 0.41) with power (1 - β) set at 0.80, and α = 0.05. The fear-potentiated startle (FPS) paradigm was performed to assess the short-term diet effects on cued fear conditioning and fear extinction learning at PND 38–41 (ADOL group) and PND 88–91 (ADUL group). We measured anxiety-like responses in the elevated plus maze (EPM) at PND 42 (ADOL) and PND 92 (ADUL). All the rats were euthanized 48 h following EPM testing. The rats were allowed to consume the custom diets until completion of the study at PND 44 (ADOL) and PND 94 (ADUL). Figure 1 summarizes the timeline of experimental procedures and behavioral tests.

### Acoustic Startle Reflex

The ASR experiments were performed using the SR-Lab acoustic chambers (San Diego Instruments, San Diego, CA, United States). ASR magnitudes were measured by placing animals in startle enclosures with sensors (piezoelectric transducers) that convert small movements to voltage. Thus, the magnitude of the change in voltage represents the size of the ASR. Acoustic stimuli intensities and response sensitivities were calibrated before commencing the experiments. The ASR protocol has been previously described by our group (Kalyan-Masih et al., 2016; Vega-Torres et al., 2018). Briefly, experimental sessions were 22 min long and started with a 5-min habituation period (background noise = 60 dB). The rats were then presented with a series of 30 tones (10 tones at each 105 dB) using a 30-s intertrial interval (ITI). The acoustic stimuli had a 20-ms duration. Subsequently, the rats were returned to their home cages. Enclosures were thoroughly cleaned and dried following each session. Averaged ASR magnitudes were normalized by weight to eliminate confounding factors associated with body weight (weight-corrected ASR = maximum startle magnitude in mV divided by body weight at testing day) (Gogos et al., 1999; Elkin et al., 2006; Grimsley et al., 2015; Kalyan-Masih et al., 2016; Vega-Torres et al., 2018). Baseline ASR responses were measured before commencing the dietary manipulations (PND 30, ADOL group; PND 80, ADUL group).

### Fear Potentiated Startle

The fear potentiated startle (FPS) protocol was adapted from Davis (2001) and detailed in our previous studies (Vega-Torres et al., 2018). Each FPS session started with a 5-min acclimation period (background noise = 60 dB). During the first session of the paradigm (fear training), the rats were trained to associate a light stimulus [conditioned stimulus (CS)] with a 0.6-mA footshock [unconditioned stimulus (US)]. The conditioning session involved 10 CS + US pairing presentations. During each CS + US presentation, the light (3,200 ms duration) was paired with a coterminating footshock (500 ms duration). Light-shock pairings were presented in a quasi-random manner (ITI = 3–5 min). Cued fear acquisition was measured 24 h later. During the second session (fear learning testing; pre-extinction FPS), the rats were first presented with 15 startle-inducing tones (*leaders*; 5 each at 90, 95, and 105 dB) delivered alone at 30 s ITI. Subsequently, the rats were presented with 60 test trials. For half of these test trials, a 20-ms tone was presented alone (tone-alone trials; 10 trials for each tone: 90, 95, and 105 dB). For the other half, the tone was paired with a 3,200-ms light (light + tone trials; 10 trials for each pairing: 90, 95, and 105 dB). The 60 trials were divided into 10 blocks of 6 test trials each which included three tone-alone trials and three light + tone trials. To conclude the testing session, the rats were presented with 15 startle-inducing tones (*trailers*; 5 each at 90, 95, and 105 dB) delivered at 30 s ITI. Trials in this session were presented in a quasi-random order (ITI = 30 s). The startle-eliciting tones had a 20-ms duration. One day after fear conditioning testing, the rats were exposed to a single extinction-training session. The extinction training session consisted of 30 CS alone presentations (light without shock or noise bursts) with a duration of 3,700 ms (ITI = 30 s). One day after fear extinction training, we determined fear extinction acquisition using the same FPS session that was used to measure fear acquisition. It is noteworthy that, in this study, we shortened the fear extinction training protocol to a single session as opposed to three sessions in our previous report in adult rats (Vega-Torres et al., 2018). A single extinction training session enables accurate and precise fear extinction measurements without flooring effects in adolescent Lewis rats. We assessed fear learning and fear extinction learning by comparing the startle amplitude from light + tone trials [conditioned + unconditioned stimulus (CS + US)] relative to tone alone trials (US). FPS data were reported as the proportional change between US and CS + US {%FPS = [(light + tone startle) - (tone-alone startle)]/(tone-alone startle) × 100} (Walker and Davis, 2002). Fear recovery, a proxy for fear extinction memory retention, was scored as the ratio between the FPS value from fear extinction and fear learning testing sessions [%fear recovery = (FPS extinction/FPS learning) × 100].

### Elevated Plus Maze

The near-infrared (NIR)-backlit EPM consisted of two opposite open arms (50.8 × 10.2 × 0.5 cm) and two opposite enclosed arms (50.8 × 10.2 × 40.6 cm) (Med Associates Inc., Fairfax, VT, United States). The arms were connected by a central 10 × 10 cm square-shaped area. The maze was elevated 72.4 cm from the floor. Behaviors were recorded in a completely dark room. The rats were placed on the central platform facing an open arm and allowed to explore the EPM for 5 min. The apparatus was cleaned after each trial (70% ethanol, rinsed with water, and then dried with paper towels). The behaviors were monitored via a monochrome video camera equipped with an NIR filter and recorded and tracked using Ethovision XT (Noldus Information Technology, Leesburg, VA). In rats, changes in the percentage of time spent on the open arms (OAs) indicate changes in anxiety (Pellow et al., 1985), and the number of closed arm (CA) entries is the best measure of locomotor activity (File, 1992). These data are used to calculate the anxiety index (Cohen et al., 2012; Contreras et al., 2014; Kalyan-Masih et al., 2016):

Anxiety⁢Index =1-{[(OAcumulativeduration/totaltestduration)+(OAentries/totalnumberofentriestoCA+OA)]/2}

### c-Fos Free-Floating Immunofluorescence

A separate cohort of adolescent and adult rats was exposed to electric footshock stress using the same paradigm employed during fear conditioning. This session consisted of 10 CS + US pairing presentations with a light (3,200 ms duration) paired with a coterminating footshock (0.6 mA footshock; 500 ms duration). Light-shock pairings were presented in a quasi-random manner (ITI = 30 s–2 min). One hour after the session, the rats were sacrificed via transcardiac perfusion with 4% paraformaldehyde (PFA) using the Perfusion Two^TM^ Automated Pressure Perfusion System (Leica Biosystems, Buffalo Grove, IL) (*n* = 12; 3 rats per group). The brains were removed from the cranial vault 4 h after fixation and postfixed in 4% PFA for 24 h. The brains were then washed with phosphate-buffered saline (PBS) and cryoprotected with sucrose (30%) for 12–16 h at 4°C prior to embedding in Tissue-Tek^®^ O.C.T.^TM^ compound (Sakura, Torrance, CA, United States).

#### Tissue Sampling

Brain tissue was cut coronally at 25 μm thickness. All immunohistochemical techniques were performed on free-floating sections. Based on the Paxinos and Watson rat brain atlas (from bregma: + 3.20 mm to + 2.20 mm), 10 sections were chosen at a 75-μm interval (one of every four sections) covering a total 1,000 μm of area containing medial prefrontal cortex (Paxinos and Watson, 2006). An additional 13 sections were cut and chosen in the same manner (from bregma: −2.30 to −3.60 mm) covering a total 1,300 μm of area containing the amygdala.

#### c-Fos Immunofluorescence

The free-floating tissue sections were washed with PBS and then incubated for blocking with permeable buffer (0.3% Triton-X 100 in PBS) containing 10% normal goat serum for 50 min. The sections were incubated overnight at 4°C in the primary antibodies against c-Fos (1:6,400; catalog no. 2250S; Cell Signaling Technology, Danvers, MA) and then incubated with the secondary Alexa Fluor^®^ 448 (1:800; catalog no. 4412; Cell Signaling Technology). The slices were then rinsed with PBS, mounted on microscope slides with ProLong^TM^ Gold Antifade mountant containing 4′,6-diamidino-2-phenylindole (DAPI) (Molecular Probes, Eugene, OR) and cover-slipped.

#### Analyses

The mPFC and amygdala were identified as region of interest (ROI) based on common histological landmarks (Paxinos and Watson, 2006). We used randomization of location and orientation within navigation windows to sample within the mPFC and amygdala. We sampled from infralimbic and prelimbic cortices of the mPFC. Within the amygdala, the anterior basolateral amygdaloid nucleus (BL), the posterior basomedial amygdaloid nucleus (BM), and the ventromedial lateral amygdaloid nucleus (L) were analyzed. c-Fos-positive cells were counted within an unbiased counting frame using a cell count software (Keyence Corp. of America, Itasca, IL, United States). A Keyence Biorevo BZ-9000 All-In-One Fluorescence Microscope (Keyence) equipped with a Nikon CFI Plan Apo λ20X objective (Nikon, Melville, NY, United States) was used to collect *z*-stacks (numerical aperture, 0.75; working distance, 1.0 mm). Digitalization and image quantification were carried out by blinded observers. Cells identified as c-Fos positive after threshold correction were quantified automatically using the Macro Cell Count Software (batch image analysis tool from Keyence). For immunofluorescence analyses, a minimum of five images per area per animal were used (depending on the size of the ROI). Image analyses were averaged per ROI, and the total sample number used for statistical analysis equaled the number of animals used.

### Real-Time Quantitative Polymerase Chain Reaction

Rats were euthanized with Euthasol (150 mg/kg, i.p.; Virbac, Fort Worth, TX, United States) and quickly perfused transcardially with PBS to remove residual blood from the brain capillaries. Following the perfusion procedure, the prefrontal cortex was isolated from a subcohort of rats that underwent behavioral testing (*n* = 6–8/group). Total RNA was extracted using Trizol (Invitrogen Life Technologies, Carlsbad, CA, United States). The only change to the recommended protocol was using 1-bromo-3-chloropropane (BCP, 0.1 ml per 1 ml of Trizol) instead of chloroform. BCP was obtained from the Molecular Research Center (Cincinnati, OH, United States). RNA concentration was determined on a NanoDrop spectrophotometer (Thermo Fisher Scientific, Waltham, MA, United States). In our hands, this protocol results in an average RNA purity between 1.9 and 2.0, 260/280 ratio. We used 1 μg of the total RNA for complementary DNA (cDNA) synthesis (iScript cDNA Synthesis Kit, catalog no. 170-8891, Bio-Rad Laboratories, Hercules, CA, United States). cDNA synthesis protocol was performed according to the manufacturer’s instructions. The total volume of the cDNA synthesis reaction mixture was 20 μl (4 μl, iScript reaction mix; 1 μl, iScript reverse transcriptase; 15 μl nuclease-free water, and 1 μg of RNA). After completion of cDNA synthesis, 80 μl of nuclease-free water was added to dilute the 20 μl of synthesized cDNA. The cDNA was amplified by PCR using the primer sets described in Table 2. Real-time PCR amplification and analyses were carried out on the CFX96 Real-Time PCR Detection System (Bio-Rad Laboratories, Hercules, CA, United States). Real-time quantitative PCR (qPCR) conditions were optimized, and 25-μl reactions were prepared. The PCR reactions contained: 12.5 μl of iQ SYBR Green Supermix (catalog no. 170-8882, Bio-Rad Laboratories, Hercules, CA, United States), 1 μl of a mixture of 10 μM forward/reverse primer, 6.5 μl of water, and 5 μl of the previously synthesized cDNA. The PCR protocol started with 5 min at 95°C. This was followed by 40 cycles of 15 s at 95°C for denaturation and 1 min at 60°C for annealing/extension. The relative levels of mRNA were calculated using the comparative *Ct* (crossing threshold). Each sample was normalized to its glyceraldehyde 3-phosphate dehydrogenase (GAPDH) mRNA content. Relative gene expression levels were normalized to the adolescent CD group and expressed as fold change.

**TABLE 2:** Primer sequences used in the study.

Gene (accession number)	Forward (5′–3′)	Reverse (5′–3′)
*Crhr1* (NM_030999)	CCAATCCAGCTTTCTGTCACTTA	CCGACCCGATCTTCCCAC
*Drd1* (NM_010076)	ATCGTCACTTACACCAGTATCTACAGGA	GTGGTCTGGCAGTTCTTGGC
*Drd2* (NM_012547)	AGACGATGAGCCGCAGAAAG	GCAGCCAGCAGATGATGAAC
*Fkbp51* (NM_001012174)	TGGTTGAGCAGGGAGAAGAT	GCCAAGGCTAAAGACAAACG
*Fkbp52* (NM_001191863)	ACACTGGCTGGCTGCTAGAT	ATTTGGGGGAATCTTTGGAG
*Nr3c1* (M14053)	CTTGAGAAACTTACACCTCGATGACC	AGCAGTAGGTAAGGAGATTCTCAACC
*Gapdh* (NM_017008)	AGTTCAACGGCACAGTCAAG	GTGGTGAAGACGCCAGTAGA

### Glucose Measurements

Blood glucose levels were measured in a subcohort of rats that underwent behavioral testing. The rats’ tail tip was cut under anesthesia before inducing euthanasia (*n* = 6–8/group; same animals used for qRT-PCR analyses). Blood glucose levels were measured using a glucometer (OneTouch UltraMini, LifeScan).

### Corticosterone Measurements

We induced euthanasia in the same subcohort of rats used to collect molecular data (Euthasol; 150 mg/kg, i.p.). Blood collected through cardiac puncture. Blood samples were collected into ethylenediaminetetraacetic acid (EDTA)-coated tubes. Subsequently, the samples were centrifuged at 1,000 rpm for plasma fraction collection. Measurement of circulating levels of corticosterone (CORT) was done using an ELISA kit (no. ADI-900-097) from Enzo Life Sciences (Farmingdale, NY) according to the manufacturer’s instructions. Samples were diluted with kit assay buffer (1:4 dilutions) and ran in triplicates. Absorbance was measured at 405 nm with 570 nm correction using SpectraMax i3X detection platform (Molecular Devices, Sunnyvale, CA, United States). Concentrations for each sample were determined as the percentage bound using a standard curve with detection range between 20,000 and 31.25 pg/ml. Calculated values are reported as picograms of corticosterone per milliliter.

### Statistical Analysis

We analyzed the data using GraphPad Prism version 8.0. Shapiro–Wilk statistical analyses were used to determine sample distribution. The Brown–Forsythe test was used to test for the equality of group variances. When appropriate, two-way analysis of variance (ANOVA) was used to examine the effect of the diet type, age, and interaction between factors on outcomes measures. Multiple comparisons were made using Dunnett’s (following Welch’s ANOVA) or Sidak’s (repeated measures two-way ANOVA) tests. The ROUT method was used to investigate outliers. We considered differences to be significant if *p* < 0.05. The data are shown as the mean ± standard error of the mean (SEM). We also conducted a *post hoc* power analysis with the *G^∗^Power* program (Faul et al., 2007). For two-way ANOVA behavioral analyses, the statistical power (1 - β) for data including the four study groups was 0.79 for detecting a medium size effect (*d* = 0.41), whereas the power exceeded 0.99 for the detection of a large effect size (*d* = 0.8). This indicates that this study is adequately powered at or above a moderate size level (*d* = 0.4). Therefore, if chosen at random, the probability that adolescent rats that consumed the WD described here will exhibit alterations in fear-related behaviors relative to controls is 0.61. With an alpha = 0.05 and power = 0.80, *post hoc* sensitivity power analyses revealed a medium size effect (*d* = 0.55) for molecular experiments (glucose, corticosterone, and mRNA level measurement). With similar alpha and power, sensitivity analyses demonstrated a large size effect (*d* = 0.92) for cFos histological experiments. These results indicate that the molecular studies were sensitive for effect sizes between 0.55 and 0.92, for a level of power equal to 0.80.

## Results

### Bodyweight and Food Consumption

This study investigated early brain and behavior responses triggered by a short-term exposure to an obesogenic diet (Figure 1A). Body weight and caloric intake were measured weekly. We found that short-term exposure to the obesogenic WD did not alter body weights in adolescent or adult rats. Repeated measures two-way ANOVA revealed a significant effect of week [*F*_(__1_._21_,_25_._35__)_ = 395.9, *p* < 0.0001] but no diet [*F*_(__1_,_21__)_ = 0.24, *p* = 0.63] or interaction [*F*_(__2_,_42__)_ = 0.88, *p* = 0.42] effects on body weight for adolescent rats (Figure 1B). Analyses showed a significant effect of week [*F*_(__1_._42_,_31_._14__)_ = 677.8, *p* < 0.0001] but no diet [*F*_(__1_,_22__)_ = 3.2, *p* = 0.09] effects on body weight in adult rats. Interestingly, we found significant interaction between diet and week in adult rats [*F*_(__2_,_44__)_ = 17.91, *p* < 0.0001] (Figure 1C). No significant interaction [*F*_(__1_,_36__)_ = 1.15, *p* = 0.29] or main effects of the diet [*F*_(__1_,_36__)_ = 0.04, *p* = 0.83] and age [*F*_(__1_,_36__)_ = 1.76, *p* = 0.19] were observed for circulating blood glucose levels at endpoint.

In agreement with previous findings, we found significant changes in caloric intake in the rats that consumed the obesogenic diet. In adolescent rats, we found a significant effect of week [*F*_(__1_,_10__)_ = 58.66, *p* < 0.0001] and diet [*F*_(__1_,_10__)_ = 17.92, *p* = 0.002], while no significant interactions [*F*_(__1_,_10__)_ = 1.52, *p* = 0.25] on caloric intake (Figure 1D). Sidak’s *post hoc* analyses showed that differences in caloric intake were statistically significant at week 1 (22% increase; *p* = 0.003) and week 2 (23% increase; *p* = 0.0005) when comparing CD and WD adolescent groups. Similarly, in adult rats, we found a significant effect of week [*F*_(__1_,_10__)_ = 21.29, *p* = 0.001] and diet [*F*_(__1_,_10__)_ = 62.28, *p* < 0.0001], while no significant interactions [*F*_(__1_,_10__)_ = 1.15, *p* = 0.31] on caloric intake (Figure 1E). *Post hoc* analyses showed that differences in caloric intake were statistically significant at week 1 (32% increase; *p* < 0.0001) and week 2 (28% increase; *p* = 0.0003) when comparing CD and WD adult groups. The caloric intake of adolescent rats exposed to the WD was similar to that of adult rats (∼900 kcal/cage/week). Notably, while adolescent rats increased caloric intake during week 2 (relative to week 1), the adult rats reduced their caloric intake. While differences in caloric needs or the period required to adjust to the novel diets may explain this effect, this finding also suggests an opposite effect of footshock stress on food consumption in adolescent and adult rats undergoing a cued fear paradigm.

### Acute WD Exposure Attenuates Fear Extinction Learning in Adolescent, but Not in Adult Rats

**FIGURE 2:**
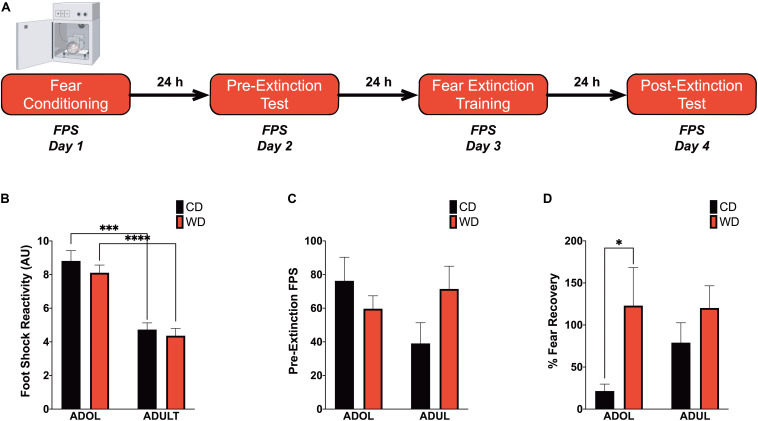
Short-term Western high-saturated fat diet (WD) exposure attenuates fear extinction in adolescent rats. **(A)** Fear-potentiated startle (FPS) paradigm. Following habituation and acclimation periods, the rats received a series of light–shock pairings (*FPS day 1, fear conditioning*). Twenty-four hours later (*FPS day 2, pre-extinction testing*), the rats were returned to the startle chambers and enclosures and presented with startle stimuli (leaders). Subsequently, rats received startle stimuli presented alone (noise-alone trial) and startle stimuli after onset of the conditioning light (light–noise trials). The rats were presented with startle-eliciting stimuli again at the end of the session (trailers). The two trial types were presented in a balanced mixed order. Twenty-four hours later (*FPS day 3, fear extinction training*), the rats were returned to the startle chambers and enclosures and presented with trials of light without shock or noise bursts. One day after fear extinction training (*FPS day 4, postextinction FPS*), we determined fear extinction acquisition and retention using the same FPS session that was used to measure fear learning. **(B)** Average pre-extinction FPS responses are similar between groups (*p* > 0.05; *n* = 9–12 rats/group). **(C)** Average post-extinction FPS responses (*p* > 0.05; *n* = 9–12 rats/group). **(D)** Average percent change in FPS responses between testing sessions as an index of fear recovery following fear extinction training. Adolescent (*ADOL*) WD rats exhibited significantly more FPS than ADOL CD rats, indicating disrupted fear extinction retention (*p < 0.05; *n* = 9–12 rats/group). Error bars are SEM. ^∗∗∗^*p* ≤ 0.001 and ^****^p ≤ 0.0001, respectively.

We recently reported that chronic consumption of an obesogenic WD during adolescence leads to impairments in fear-related associative learning and extinction in adult rats (Vega-Torres et al., 2018). However, it remains unclear from our studies whether adolescence and the obesogenic diet are predisposing and precipitating factors for the reported fear impairments. This follow-up study was designed to investigate the effects of short-term exposure to a WD on cued fear conditioning and fear extinction learning before the onset of an obesogenic phenotype in adolescent and adult rats. We used the ASR baseline values to assign and match the rats in each group based on their unconditioned responses to acoustic stimulation. Welch’s ANOVA demonstrated no significant differences in baseline weight-corrected ASR responses between groups [*F*_(__3_,_24_._4__)_ = 0.21, *p* = 0.88] (figure not shown). Following the ASR-based matching, the rats were exposed to either the control or the obesogenic custom diet and fed *ad libitum* for 1 week before behavioral testing. Anxiety and fear-related responses were investigated using the fear-potentiated startle (FPS) paradigm (Figure 2A). The rats were conditioned to learn an aversive association between a light (*CS*) and a foot shock (*US*) during the first day of the FPS paradigm. ANOVA revealed significantly reduced startle responses to the footshocks in adult rats when compared to adolescent rats [*F*_(__3_,_24_._24__)_ = 21.13, *p* < 0.0001; CD rats, *p* = 0.0001; WD rats, *p* < 0.0001] (Figure 2B). Acquired fear was tested 24 h after conditioning and defined as significant differences in startle amplitudes between the US (tone alone) and the CS + US (light + tone). All groups showed significant differences between amplitudes of US and CS + US (*p* < 0.05; data not shown). We calculated the potentiation of the startle as a proxy for fear conditioning and found that 1-week WD consumption or age did not alter fear learning [*F*_(__3_,_19_._7__)_ = 1.52, *p* = 0.24] (Figure 2C). Fear extinction training and fear extinction testing were performed on days 3 and 4 of the FPS paradigm, respectively. Successful fear extinction is defined as similar startle responses when comparing the US trials to CS + US trials (Vega-Torres et al., 2018). Although FPS responses following extinction testing were similar between groups [*F*_(__3_,_19_._7__)_ = 1.78, *p* = 0.18] (figure not shown), we found heightened fear recovery in the adolescent rats that consumed the WD relative to CD ADOL rats [Welch’s *F*_(__3_,_18__)_ = 6.27, *p* = 0.004; CD, 14.9 ± 17.92 vs. WD, 123 ± 45.21; *t*_(__14_._37__)_ = 2.22, *p* = 0.04] (Figure 2D). This finding indicates that a short-term exposure to a WD is sufficient to impair fear extinction acquisition and/or retrieval in adolescent rats.

### Acute WD Exposure Enhances Startle Sensitization in Adolescent Rats

**FIGURE 3:**
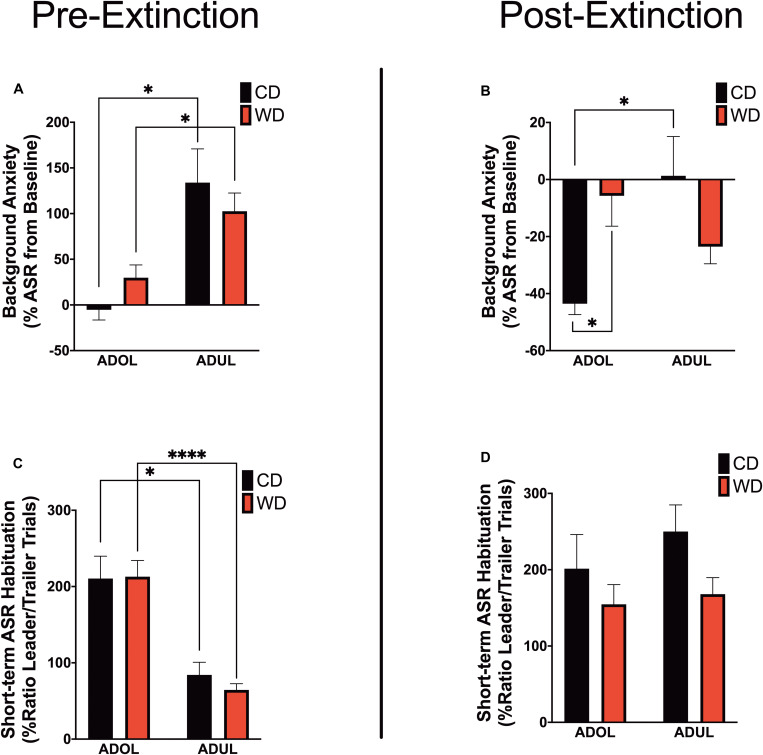
Age and diet influence acoustic startle reflex (ASR) reactivity and plasticity. **(A)** Average percent change in acoustic startle reflex (ASR) responses following fear conditioning relative to baseline. ADUL rats exhibited higher ASR responses to cued fear conditioning (*p < 0.05; *n* = 12 rats/group). **(B)** Average percent change in ASR reactivity following fear extinction training. The ADOL rats that consumed the Western-like high-saturated fat diet exhibited increased indices of anxiety relative to ADOL CD rats (*p < 0.05; *n* = 12 rats/group). Postextinction background anxiety in ADOL WD rats was similar to that observed in adult rats (*p* > 0.05). **(C)** Average pre-extinction short-term habituation of the ASR. ADUL rats exhibited higher within-session ASR responses relative ADOL rats (*p < 0.05; ****p ≤ 0.0001; *n* = 11–12 rats/group). **(D)** All groups exhibited similar short-term habituation of the ASR following fear extinction (*p* > 0.05; *n* = 10–12 rats/group). Error bars are SEM.

We reported that chronic consumption of an obesogenic WD during adolescence enhances both pre- and postextinction background anxiety (BA), as evaluated by changes in ASR responses following cued fear conditioning in adult rats (Vega-Torres et al., 2018). Here, we demonstrate that adult rats exhibited greater background anxiety before extinction training [Welch’s *F*_(__3_,_23_._3__)_ = 9.84, *p* = 0.0002; Dunnett’s *post hoc* showed an age effect for both the CD (*p* = 0.02) and WD (*p* = 0.04) rats] (Figure 3A). Notably, we found higher background anxiety in the adolescent rats that consumed the WD relative to CD adolescent rats following fear extinction training [*F*_(__3_,_21_._2__)_ = 7.16, *p* = 0.002; CD ADOL vs. WD ADOL *post hoc p* = 0.02; CD ADOL vs. CD ADUL *post hoc p* = 0.04] (Figure 3B), supporting an anxiogenic effect of the WD. Short-term habituation of the ASR measures non-associative learning and is determined using the ratio percentage from leader trials to trailer trails within each FPS testing session. We found reduced habituation of the ASR in the adult rats when compared to adolescents before fear extinction training [*F*_(__3_,_19_._7__)_ = 18.87, *p* < 0.0001; Dunnett’s test revealed an age effect for both diet groups CD (*p* = 0.05) and WD (*p* < 0.0001)] (Figure 3C), suggesting heightened sensitization to footshocks in adult rats. The effect of age in the short-term habituation of the ASR was absent following extinction training [*F*_(__3_,_22_._4__)_ = 1.74, *p* = 0.18] (Figure 3D). The long-term habituation of the ASR was determined using the ratio percentage from trailer trials to trailer trials between FPS testing sessions. Analyses revealed that adult rats exhibited reduced long-term habituation of the ASR [*F*_(__3_,_20_._8__)_ = 9.58, *p* = 0.0004; Dunnett’s test revealed an age effect for both diet groups CD (*p* = 0.02) and WD (*p* < 0.037)] (figure not shown).

### Age Increases Locomotor Activity in the Elevated Plus Maze

**FIGURE 4:**
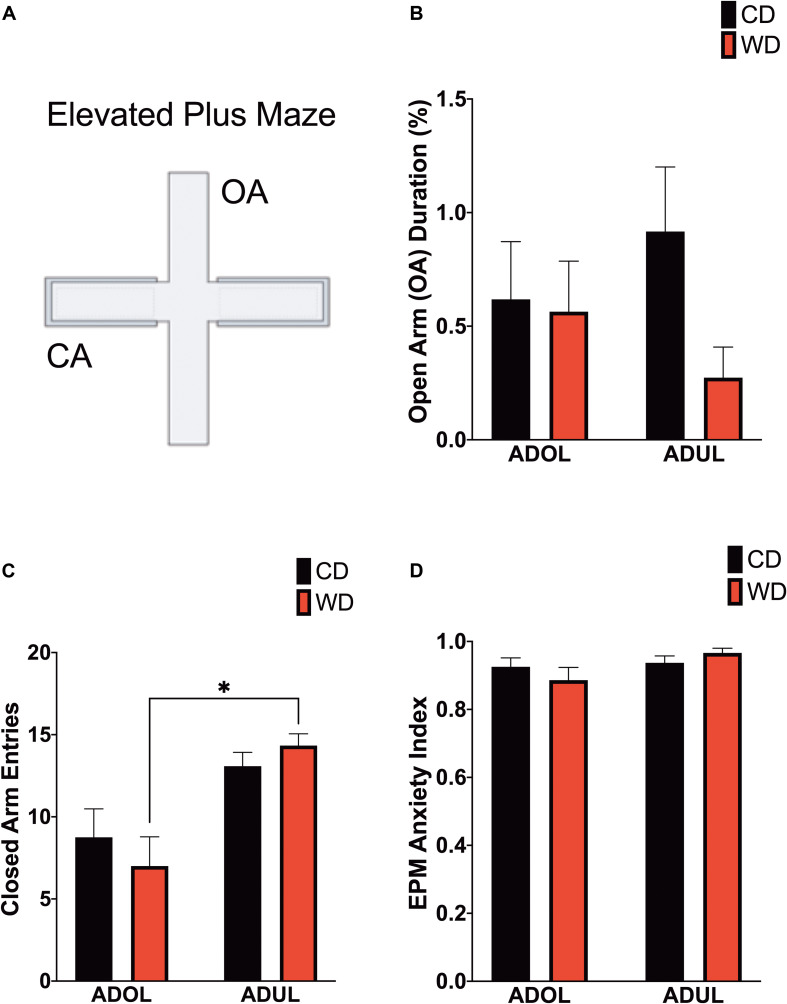
Age influences locomotor activity in the elevated plus maze (EPM). **(A)** Schematic representation of the EPM. **(B)** Average time spent in open arms (expressed as percentage of total time in maze) was similar between groups (*p* > 0.05; *n* = 10–12 rats/group). **(C)** Average entries to closed arms. ADUL Western-like high-saturated fat diet (WD) rats exhibited higher ambulation when compared to ADOL WD rats (^∗^*p* < 0.05; *n* = 10–12 rats/group). **(D)** There was no significant difference in the anxiety index (*p* > 0.05; *n* = 10–12 rats/group). Error bars are SEM.

Given that footshock stress and obesogenic diet consumption modulate anxiety-like behavior, we carried out analyses of behavioral responses in the EPM 1 day following fear extinction testing (Figure 4A). There was no difference in the duration in the open arms [*F*_(__3_,_21_._6__)_ = 1.6, *p* = 0.21] (Figure 4B). The locomotor activity, reflected by the number of crossings between closed arms, was different among groups [*F*_(__3_,_23_._19__)_ = 6.51, *p* = 0.002] (Figure 4C). Ambulation was higher in WD ADUL rats than in WD ADOL rats (adjusted *p* = 0.001). It is noteworthy to mention that we found very robust avoidance responses and anxiety-like behaviors relative to previous studies, suggesting that the fear conditioning paradigm used in this study is anxiogenic. The analysis of the anxiety index, which incorporates several behavioral measures in the maze, revealed no significant differences between groups [*F*_(__3_,_22_._98__)_ = 1.72, *p* > 0.05] (Figure 4D), possibly reflecting a ceiling effect.

### Acute WD Exposure During Adolescence Attenuates Footshock Stress-Induced c-Fos Expression in the Basomedial Amygdala

**FIGURE 5:**
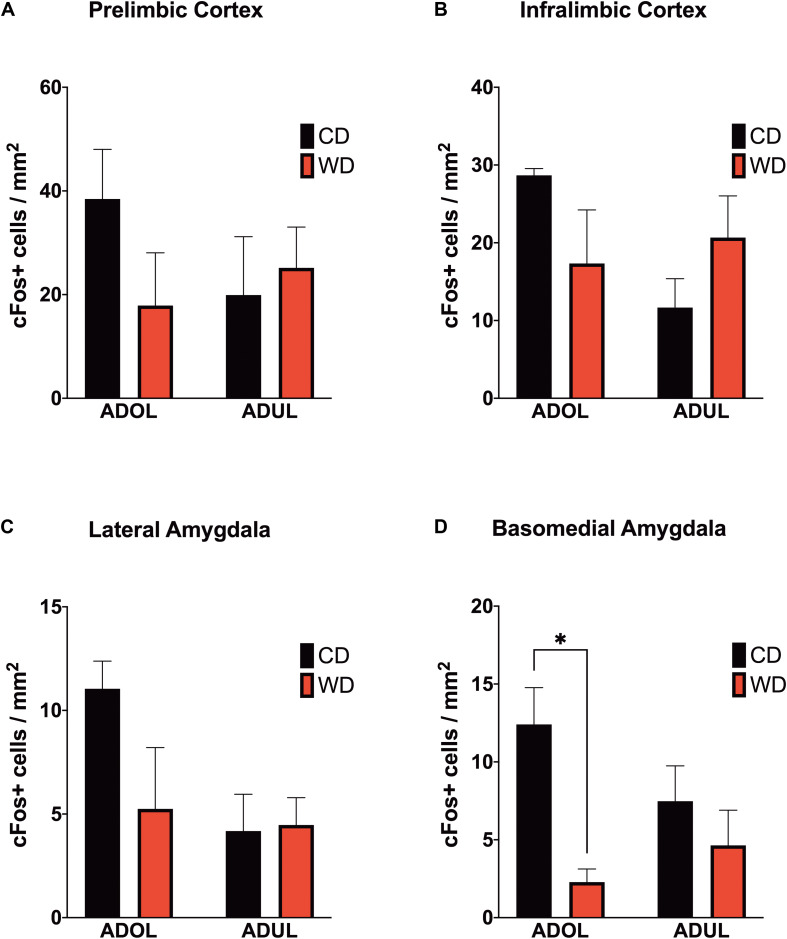
Western-like high-saturated fat diet (WD) consumption during adolescence attenuates neuronal activation to footshock stress in the basomedial nuclei of the amygdala. Average expression of the immediate-early gene c-Fos in **(A)** prelimbic and **(B)** infralimbic medial prefrontal cortex, and in the **(C)** lateral and **(D)** basomedial nuclei of the amygdala. ADOL WD rats showed attenuated footshock-induced c-Fos expression in BMA neurons relative to ADOL CD rats (^∗^*p* < 0.05; *n* = 3 rats/group). Error bars are SEM.

We reported that the consumption of an obesogenic diet has detrimental consequences for the structural integrity of mPFC–amygdala circuits implicated in emotion regulation and fear (Vega-Torres et al., 2018). To further characterize neural substrates impacted by obesogenic diets, we evaluated neuronal activation in mPFC and amygdala regions implicated in fear. We mapped c-Fos expression 1 h after the rats were exposed to a cued fear conditioning session. We found no significant main effects of diet [*F*_(__1_,_8__)_ = 0.61, *p* = 0.45], age [*F*_(__1_,_8__)_ = 0.33, *p* = 0.58], or interactions [*F*_(__1_,_8__)_ = 1.74, *p* = 0.22] on footshock-induced c-Fos expression in the prelimbic region of the mPFC (Figure 5A). Similarly, the diet type [*F*_(__1_,_8__)_ = 0.06, *p* = 0.81], age [*F*_(__1_,_8__)_ = 2.06, *p* = 0.18], and interactions between factors [*F*_(__1_,_8__)_ = 4.55, *p* = 0.06] did not have a significant effect on c-Fos protein levels in the infralimbic region of the mPFC (Figure 5B). The basolateral, basomedial, and lateral amygdaloid nuclei were also assessed for cued fear conditioning-induced c-Fos expression. While analyses revealed no significant effects of the diet, age, and interactions in c-Fos expression in the basolateral [diet: *F*_(__1_,_8__)_ = 1.20, *p* = 0.31; age: *F*_(__1_,_8__)_ = 0.11, *p* = 0.76; interaction: *F*_(__1_,_8__)_ = 0.23, *p* = 0.65] (figure not shown) and lateral amygdaloid nuclei [diet: *F*_(__1_,_8__)_ = 1.97, *p* = 0.20; age: *F*_(__1_,_8__)_ = 3.78, *p* = 0.09; interaction: *F*_(__1_,_8__)_ = 2.40, *p* = 0.16] (Figure 5C), we found that the obesogenic diet reduced c-Fos expression levels in the basomedial nucleus of the amygdala [diet: *F*_(__1_,_8__)_ = 10.15, *p* = 0.01; age: *F*_(__1_,_8__)_ = 0.4, *p* = 0.55; interaction: *F*_(__1_,_8__)_ = 3.21, *p* = 0.11] (Figure 5D). Sidak’s *post hoc* analysis revealed that acute exposure to the WD during adolescence led to a significant reduction in basomedial amygdala c-Fos expression relative to control rats (adjusted *p* = 0.02).

### Age and Short-Term Obesogenic Diet Exposure Modulate the Levels of Critical Fear-Modulating Biomarkers

**FIGURE 6:**
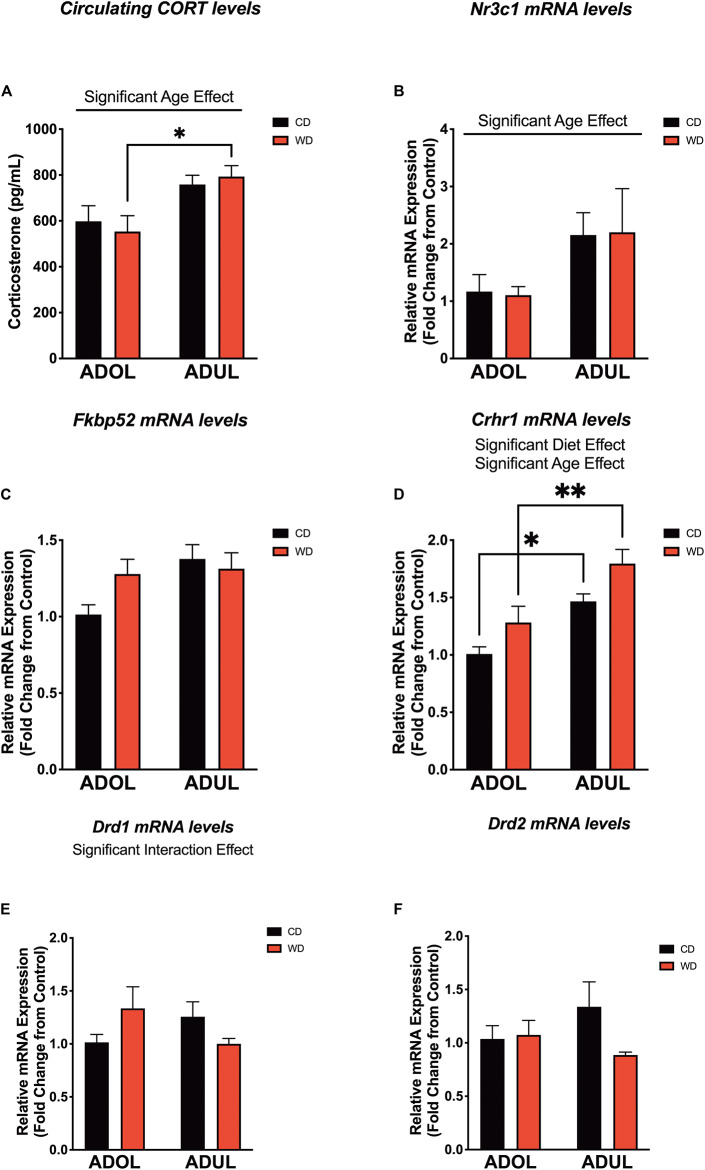
Age and short-term exposure to obesogenic diet increase stress and fear-related biomarkers in the medial prefrontal cortex. **(A)** Age increased plasma corticosterone levels in Western-like high-saturated fat diet (WD) rats (*p = 0.01; *n* = 6–8 rats/group). **(B)** Similarly, age had a significant impact on the messenger RNA levels of glucocorticoid receptor gene (*Nr3c1*) (*p = 0.03; *n* = 8 rats/group). **(C)** Age and diet had no effects on messenger RNA (mRNA) levels of FK506-binding protein 2 (*Fkpb52*) (*p* > 0.05; *n* = 8 rats/group). **(D)** Both age (*p* = 0.0002) and diet (*p* = 0.0093) had robust effects in the expression of the corticotropin-releasing hormone receptor 1 (*Crhr1*) mRNA. **(E)** There was a significant interaction between age and the obesogenic diet on the expression of the dopamine receptor D1 (*Drd1*) (*p* < 0.05; *n* = 7–8 rats/group). **(F)** There were no significant differences in the mRNA levels of the dopamine receptor D2 (*Drd2*) (*p* > 0.05; *n* = 7–8 rats/group). *p < 0.05; **p < 0.01. Data represents mean ± SEM.

We evaluated the levels of critical fear-associated biomarkers to gain insights on putative early (mal)adaptive mechanisms impacted by a short-term exposure to the obesogenic diet. We found a significant effect of age on plasma corticosterone (CORT) levels [diet: *F*_(__1_,_24__)_ = 0.007, *p* = 0.93; age: *F*_(__1_,_24__)_ = 13.22, *p* = 0.001; interaction: *F*_(__1_,_24__)_ = 0.51, *p* = 0.48] (Figure 6A). The adult rats exposed to the short-term WD had significantly higher CORT levels when compared to the adolescent rats that consumed the same diet (*p* = 0.01). Consistent with this finding, age had a significant impact on the mRNA expression levels of the glucocorticoid receptor gene (*Nr3c1*) in the medial prefrontal cortex, with adult rats exhibiting higher Nr3c1 mRNA levels relative to adolescent rats [diet: *F*_(__1_,_20__)_ = 0.0002, *p* = 0.98; age: *F*_(__1_,_20__)_ = 5.14, *p* = 0.03; interaction: *F*_(__1_,_20__)_ = 0.01, *p* = 0.9] (Figure 6B). Although the adult rats showed a trend for higher mRNA levels of the glucocorticoid receptor chaperones, *Fkbp51* (figure not shown) and *Fkbp52* (Figure 6C), analyses showed no significant effects of the diet type, age, and interactions between factors [for FKBP5-1, diet: *F*_(__1_,_20__)_ = 0.34, *p* = 0.56; age: *F*_(__1_,_20__)_ = 1.97, *p* = 0.17; interaction: *F*_(__1_,_20__)_ = 0.05, *p* = 0.82; for FKBP5-2, diet: *F*_(__1_,_19__)_ = 0.65, *p* = 0.42; age: *F*_(__1_,_19__)_ = 3.60, *p* = 0.07; interaction: *F*_(__1_,_19__)_ = 2.32, *p* = 0.14].

The corticotropin-releasing hormone receptor 1 (*Crhr1*) is abundantly expressed in the mPFC and serves crucial roles in the regulation of stress and fear responses. We found that the rats that were exposed to the short-term obesogenic diet had higher Crhr1 mRNA levels in the mPFC relative to controls [diet: *F*_(__1_,_20__)_ = 8.28, *p* = 0.009] (Figure 6D). Adult rats showed increased Crhr1 mRNA levels in the mPFC relative to adolescent rats [age: *F*_(__1_,_20__)_ = 21.58, *p* = 0.0002; interaction: *F*_(__1_,_20__)_ = 0.07, *p* = 0.79]. Sidak’s *post hoc* analysis revealed that adult rats in both CD (*p* = 0.01) and WD (*p* = 0.004) groups had significantly higher Crhr1 mRNA levels in the PFC when compared to adolescent rats. Previous studies indicate the active involvement of dopaminergic receptors in diet-induced obesity and fear-related behaviors. Of interest, evidence indicates that dopamine receptors are under regulatory actions of corticotropin-releasing hormone (CRH) and dictate fear conditioning and fear extinction responses. Thus, we aimed to determine the effects the obesogenic diet, age, and interactions on the mRNA levels of dopamine receptors 1 and 2. While diet [*F*_(__1_,_19__)_ = 0.05, *p* = 0.82] and age [*F*_(__1_,_19__)_ = 0.11, *p* = 0.73] did not have significant effects on the mRNA expression levels of the dopamine receptor 1 (*Drd1*), we found a significant interaction between these factors [*F*_(__1_,_19__)_ = 4.39, *p* = 0.04] (Figure 6E). While the two-way ANOVA revealed a significant global interaction effect for Drd1, *post hoc* analysis did not show significant differences between groups. It is likely that this discrepancy between the ANOVA and *post hoc* analysis may be related to the weakly global effect found (*p* = 0.04) and/or associated with the conservative Sidak’s multiple comparison test used in this study. We found no significant effects of the diet [*F*_(__1_,_18__)_ = 1.71, *p* = 0.20], age [*F*_(__1_,_18__)_ = 0.13, *p* = 0.72], or interactions [*F*_(__1_,_18__)_ = 2.38, *p* = 0.14] on the expression of Drd2 mRNA levels in the mPFC (Figure 6F). Given the similar pattern of decreased mRNA expression of dopamine receptors in adult rats, we decided to collapse the data into a within-subjects analysis (with diet and receptor gene type as factors) to add power and determine the effects of the obesogenic diet. Interestingly, analyses revealed a significant global effect of the short-term exposure to the obesogenic diet in reducing Drd1 and Drd2 mRNA levels in the mPFC of adult rats [diet: *F*_(__1_,_18__)_ = 5.40, *p* = 0.03; receptor gene type: *F*_(__1_,_18__)_ = 0.011, *p* = 0.92; interaction: *F*_(__1_,_18__)_ = 0.41, *p* = 0.53]. On the other hand, there was no significant effect of the diet [*F*_(__1_,_19__)_ = 1.54, *p* = 0.23], dopamine receptor gene type [*F*_(__1_,_19__)_ = 0.69, *p* = 0.43], or interactions [*F*_(__1_,_19__)_ = 0.96, *p* = 0.34] in the adolescent rats.

## Discussion

Childhood trauma survivors who suffer from PTSD are at high risk for developing obesity and metabolic disorders (Llabre and Hadi, 2009; Roenholt et al., 2012; Assari et al., 2016; Brewerton and ONeil, 2016; Ramirez and Milan, 2016). We reported evidence in support of these observations while demonstrating a novel directionality in the relationship between early-life posttraumatic stress reactivity and diet-induced obesity (DIO) (Kalyan-Masih et al., 2016; Vega-Torres et al., 2018). Our findings indicate that the consumption of an obesogenic Western-like high-saturated fat/high-sugar diet (WD) during adolescence heightens stress reactivity while altering key substrates implicated in PTSD (Kalyan-Masih et al., 2016; Vega-Torres et al., 2018).

Although the long-term impact of DIO on emotion regulation is becoming clear, mechanistic studies are confounded by the multiple complications associated with obesity. In this study, we expanded our previous observations and investigated the impact of a short exposure to a WD, including groups exposed to the WD during adolescence and adulthood. Importantly, we tested the acute WD effects, relative to an ingredient-matched low-fat semipurified control diet, on cued fear conditioning and fear extinction using the FPS paradigm. The primary findings of this study are the following: (a) short exposure to a WD impairs cued fear extinction memory retention in adolescent rats; (b) short-term WD consumption attenuates basomedial amygdala activation to a fear conditioning paradigm; (c) short-term WD consumption increases CRHR1 mRNA expression levels in the medial prefrontal cortex; and (d) adult rats exhibit markers of heightened HPA axis tone and emotional reactivity relative to adolescent rats. Altogether, this follow-up study provides supportive evidence that behavioral and molecular substrates implicated in fear are selectively vulnerable to the consumption of an obesogenic WD during adolescence. Our findings also serve to emphasize the caution that must be exercised when interpreting experimental outcomes when unmatched diets are used as controls in DIO studies.

### Short-Term WD Consumption Attenuates Fear Extinction Learning

An important finding of this study is that short-term WD consumption was sufficient to attenuate extinction learning of cued fear. Fear extinction has been characterized as an active form of inhibitory learning that allows for the adaptive regulation of conditioned fear responses (Myers and Davis, 2002). The inability to consolidate extinction memory and inhibit conditioned fear under safe conditions underlies some of the hallmark symptoms of anxiety and stress-related disorders (Milad et al., 2009; McGuire et al., 2016; Waters and Pine, 2016). It is now recognized that cognition, attention, mood, and anxiety disorders have a nutritional component or are promoted by poor dietary habits. Our results are consistent with evidence showing that the consumption of obesogenic high-fat diets can attenuate cognitive functions in humans and rodents in as short as 3–9 days (Kanoski and Davidson, 2010; Edwards et al., 2011; Holloway et al., 2011; Beilharz et al., 2014, 2016; Sobesky et al., 2016; Khazen et al., 2019). The findings of this study indicate that the adverse effects of consuming obesogenic foods expand to additional cognitive domains regulating emotional memories and fear. This is in agreement with new studies showing that adolescent rats that consume high-fat/high-sugar diets exhibit delayed spontaneous extinction and impaired extinction retention of fear-related behaviors (Reichelt et al., 2015; Baker et al., 2016; Vega-Torres et al., 2018). Our findings extend beyond those reported to date by showing fear extinction deficits independent of effects associated with obesity and metabolic disturbances.

Evidence supports that impairments in attention and threat discrimination may heighten the risk for anxiety and stress-related psychopathology (López-Aumatell et al., 2009a, b; Block and Liberzon, 2016). This study confirms our previous findings showing that rats that consume obesogenic diets exhibit alterations in ASR plasticity, which may result in an inaccurate assessment of the level of threat (Kalyan-Masih et al., 2016; Vega-Torres et al., 2018). Notably, we found that the ASR neural substrates impacted by the WD seem to be dependent on the age of onset of diet exposure. The adolescent rats that consumed the WD exhibited increased ASR sensitization to the footshocks. This behavioral proxy may represent maladaptive stress reactivity and anxiety (Shalev et al., 2000; Conti and Printz, 2003).

Interestingly, the adult rats that consumed the WD showed greater long-term habituation of the ASR relative to CD rats. Since sensitization and habituation of the ASR require independent neural substrates (Leaton, 1976; Koch, 1999; Pilz and Leaton, 1999), our data indicate that short exposure to WD influences different targets in adolescent and adult rats. Taken together, the ASR behavioral outcomes reported here support the notion that perturbations in attention, threat discrimination, and startle habituation may heighten vulnerability for anxiety and stress-related disorders in individuals that consume obesogenic diets.

### Short Exposure to an Obesogenic Diet Attenuates Basomedial Amygdala Activation Following Cued Fear Conditioning

Several studies indicate that the mPFC and the basolateral complex of the amygdala (BLA) are critical to the acquisition and expression of conditioned fear (Sotres-Bayon et al., 2004; Davis, 2006; Quirk et al., 2006). The highly conserved neurocircuitry connecting the mPFC and the amygdala plays a critical role in anxiety and in the extinction of fear memories (Milad and Quirk, 2002; Phelps et al., 2004; Adhikari et al., 2015; Janak and Tye, 2015) and is abnormal in PTSD patients (Gilboa et al., 2004; Koenigs and Grafman, 2009). The PFC and amygdala undergo striking structural changes during adolescence (Jalbrzikowski et al., 2017), providing a biological basis that may underlie their unique vulnerability to the disruptive effects of obesity and the consumption of diets rich in saturated fats and sugars. Paralleling clinical data in humans (Riederer et al., 2016; Geha et al., 2017), we showed that DIO rats exhibit significant and partly irreversible microstructural alterations in mPFC regions and amygdalar nuclei associated with fear learning and fear extinction (Vega-Torres et al., 2018). The impact of obesogenic diets on PFC and BLA neuroplasticity is supported by studies showing reduced dendritic spine density in the PFC (Dingess et al., 2017) and dendritic length in the basal arbors of the BLA (Janthakhin et al., 2017) in rats that consume obesogenic diets rich in fats. Together, these structural alterations may lead to anxiety (Kim and Whalen, 2009), impairments in fear processing (Poulos et al., 2009), and aberrant feeding behaviors (Land et al., 2014).

### Age Increases Anxiety and Emotional Reactivity in Lewis Rats

There is some evidence in support of the notion that limbic structures involved in higher processing of emotional cues become deficiently activated with age despite showing a higher basal level of activation (Meyza et al., 2011). The histological results presented in this study provide support to this notion by indicating different c-Fos signatures in adolescent rats, with greater activation in mPFC and amygdalar regions, relative to adult rats. This suggests that the typical age-related increases in anxiety in adult LEW rats may be related to a blunted neuroendocrine response, combined with insufficient top–down regulation of limbic regions fear and anxiety (Adhikari et al., 2015).

It is becoming increasingly clear that obesogenic diets dysregulate critical mediators of the HPA axis in rats (Auvinen et al., 2012; Abildgaard et al., 2014; Boitard et al., 2015; McNeilly et al., 2015; Kalyan-Masih et al., 2016; Sobesky et al., 2016; Vega-Torres et al., 2018; Khazen et al., 2019). The present findings seem difficult to reconcile with these reports, as they indicate that short exposure to a WD was not sufficient to increase the circulating levels of corticosterone and related neuroendocrine biomarkers in the prefrontal cortex. However, differences in the methodology (fear conditioning paradigm) and diet composition (fat content and source; purified ingredient-matched control diet) may explain this discrepancy. Nonetheless, our findings support an age-related enhancement of the HPA axis tone in LEW rats (Meyza et al., 2011).

### Age and Diet Modulate the Expression of the Corticotropin-Releasing Hormone Receptor 1

The corticotropin-releasing hormone (CRH) system has received considerable attention as a promising therapeutic target for anxiety and stress-related disorders (Bale and Vale, 2004; Binder and Nemeroff, 2010). CRH is an essential component of the behavioral and endocrine responses to stress, signaling through the stimulation of the G-protein-coupled CRH receptor 1 (CRHR1). The CRHR1 is abundantly expressed in fear-modulating corticolimbic circuits, including the mPFC (Steckler and Holsboer, 1999). Studies in humans and rodents indicate that CRH-CRHR1 hypersignaling represents a candidate mechanism for PTSD risk (Bremner et al., 1997; Rajbhandari et al., 2015; Toth et al., 2016; Jovanovic et al., 2020). CRHR1 hypersignaling alters brain structural integrity (Chen et al., 2004; Kolber et al., 2010; Toth et al., 2014) and has long-lasting consequences in stress susceptibility (Uribe-Mariño et al., 2016), mainly when it is triggered early in life (Toth et al., 2016).

Although the role of CRH in obesity is very complex, studies demonstrate that the CRH system has anorectic and thermogenic roles (Kuperman et al., 2016). CRH-CRHR1 signaling has emerged as a potential neuromodulator of food intake energy expenditure (Arase et al., 1988). Interestingly, CRHR1 is coexpressed with various metabolic receptors in corticolimbic structures (Koorneef et al., 2018), supporting an interplay between metabolism and fear. Our findings indicate that CRHR1 upregulation represents an early molecular adaptation to the obesogenic diet. Furthermore, our results provide support for further study of this signaling system as a candidate mechanism for anxiety and PTSD risk in obesity. We have demonstrated that exposure to an obesogenic diet during the critical maturational period of adolescence may permanently modify corticolimbic circuits and the response to further stressful stimuli. CRHR1 may play an integral role in the mechanisms of these long-lasting structural changes (Yang et al., 2015). Together, this study provides compelling evidence of the close relationship between energy homeostasis and the function of corticolimbic pathways modulating fear.

Extrahypothalamic CRH signaling modulates the sensitization of mesolimbic dopamine circuits to stress (Burke and Miczek, 2014). Alterations in reward-related brain areas play a major role in stress responsivity, with important implications for PTSD (Corral-Frias et al., 2013; Abraham et al., 2014; Holly and Miczek, 2016). Dopamine and its D1 and D2 receptors play critical roles in fear learning and extinction in both humans (Haaker et al., 2013, 2015) and rodents (Fadok et al., 2009a; Hitora-Imamura et al., 2015; Pignatelli et al., 2017; Zbukvic et al., 2017; Ng et al., 2018). In addition to changes in genes associated with dopamine uptake and metabolism, reduced D1 and D2 receptor expression levels have been documented in obesity (Wang et al., 2001; Huang et al., 2005; Davis et al., 2009; Alsiö et al., 2010; Johnson and Kenny, 2010; Tomasi et al., 2015; Carlin et al., 2016). Please refer to Reichelt (2016) for an influential review on this topic (Reichelt, 2016). Consistent with these studies, our findings demonstrate that 1 week in the obesogenic diet was sufficient to reduce dopamine receptor mRNA levels in the mPFC. Interestingly, this effect was only observed in adult rats, possibly reflecting the dynamic changes in the expression of these receptors across mPFC maturation (Burke and Miczek, 2014). Given the critical role of dopamine signaling in fear extinction, our findings suggest that diet-induced neuroadaptations in this system could have implications for stress-related psychopathology in obese individuals.

### Limitations and Future Studies

This study had some limitations to be addressed in future research. The results should be interpreted with caution, as results of this rat model do not necessarily directly translate to the human condition. Whereas our results are consistent with deficits in fear extinction, it is unclear from our data whether the WD effects are related to impairments in fear extinction memory acquisition, consolidation, expression, reconsolidation, and/or retrieval. Continued translational work will inform the basis of these learning and memory deficits. We analyzed mPFC markers only based on our prior studies revealing long-lasting effects of a short-term exposure to an obesogenic diet in this region and its critical role in fear extinction learning, retention, and expression (Vega-Torres et al., 2018). Future studies investigating the molecular landscape of the amygdala and other mesocorticolimbic structures would better characterize the impact of obesogenic diets in brain centers implicated with emotional regulation. Several lines of evidence demonstrate that high-fat diets and fear conditioning alter the HPA axis and dopamine function markers in the brain. Therefore, it is likely that a combination of these factors contributed to the observed differences in mRNA levels. Future studies are required to clarify the relative contribution of diet and fear conditioning to gene expression. Lastly, replication in additional rats, different rat strains, sex, and conditioning paradigms is warranted.

### Summary

This study shows that the consumption of obesogenic diets during adolescence heightens behavioral vulnerabilities associated with risk for anxiety and stress-related disorders. Given that fear extinction promotes resilience against PTSD and fear extinction principles are the foundation of psychological treatments for anxiety and stress-related disorders, understanding how obesity and obesogenic diets affect the acquisition and expression of fear extinction memories is of tremendous clinical relevance.

## Data Availability Statement

The datasets generated for this study are available on request to the corresponding author.

## Ethics Statement

The animal study was reviewed and approved by the Loma Linda University IACUC.

## Author Contributions

JF and JV-T planned the experiments, tested the data statistically, and wrote the manuscript. JV-T conducted the behavioral and molecular experiments, including ASR, FPS, and EPM. MA and RR-O performed the histological experiments and quantified c-Fos staining. AR-R contributed to the RT-qPCR studies, discussion, and the manuscript. PO-A conducted and analyzed the ELISA data. All authors contributed to the article and approved the submitted version.

## Conflict of Interest

The authors declare that the research was conducted in the absence of any commercial or financial relationships that could be construed as a potential conflict of interest.
